# Discovery of uncompetitive inhibitors of SapM that compromise intracellular survival of *Mycobacterium tuberculosis*

**DOI:** 10.1038/s41598-021-87117-x

**Published:** 2021-04-07

**Authors:** Paulina Fernández-Soto, Joshua Casulli, Danilo Solano-Castro, Pablo Rodríguez-Fernández, Thomas A. Jowitt, Mark A. Travis, Jennifer S. Cavet, Lydia Tabernero

**Affiliations:** 1 School of Biological Sciences, Faculty of Biology Medicine and Health, Manchester Academic Health Science Centre, University of Manchester, Manchester, M13 9PT UK; 2 Lydia Becker Institute for Immunology and Inflammation, University of Manchester, Manchester, UK; 3 Wellcome Centre for Cell-Matrix Research, University of Manchester, Manchester, UK

**Keywords:** Biochemistry, Enzymes, Hydrolases

## Abstract

SapM is a secreted virulence factor from *Mycobacterium tuberculosis* critical for pathogen survival and persistence inside the host. Its full potential as a target for tuberculosis treatment has not yet been exploited because of the lack of potent inhibitors available. By screening over 1500 small molecules, we have identified new potent and selective inhibitors of SapM with an uncompetitive mechanism of inhibition. The best inhibitors share a trihydroxy-benzene moiety essential for activity. Importantly, the inhibitors significantly reduce mycobacterial burden in infected human macrophages at 1 µM, and they are selective with respect to other mycobacterial and human phosphatases. The best inhibitor also reduces intracellular burden of *Francisella tularensis,* which secretes the virulence factor AcpA, a homologue of SapM, with the same mechanism of catalysis and inhibition. Our findings demonstrate that inhibition of SapM with small molecule inhibitors is efficient in reducing intracellular mycobacterial survival in host macrophages and confirm SapM as a potential therapeutic target. These initial compounds have favourable physico-chemical properties and provide a basis for exploration towards the development of new tuberculosis treatments. The efficacy of a SapM inhibitor in reducing *Francisella tularensis* intracellular burden suggests the potential for developing broad-spectrum antivirulence agents to treat microbial infections.

## Introduction

Tuberculosis (TB) is still a major global health threat with 10 million people developing active TB and over 1.5 million deaths every year^1^. Current TB treatments are long (6–9 months) with multiple drugs that cause severe hepatotoxicity and other serious side effects. In addition, the emergence of drug resistance is now a major challenge as effective treatments are lacking. Two recent new drugs, bedaquiline^2^ and delamanid^3^ were approved for the treatment of multidrug-resistant TB (MDR-TB). However, resistance to both drugs was reported in less than a year after clinical use^4,5^, and the number of cases continue to rise^6,7^. The increase of MDR-TB and extremely drug resistant TB (XDR-TB) cases underlines the need to develop new drugs with novel mechanisms of action.

Antivirulence agents differ from conventional antibiotics in that they do not target bacterial growth directly, instead they block virulence factors that cause host damage and disease^8^. Targeting bacterial virulence is a non-traditional strategy that is gaining momentum for the treatment of microbial infections^8–11^. Recent reports have confirmed the efficacy of antivirulence agents on reducing burden of *Mycobacterium tuberculosis* (Mtb), the causative agent of TB, in vitro^12–16^ and in vivo^17,18^. However, antivirulence agents have yet to be incorporated in the TB drug development pipeline^19–21^.

SapM, is a secreted alkaline phosphatase from Mtb^22^, and an attractive target to exploit antivirulence strategies. Previous studies have demonstrated that SapM is essential for arresting phagosomal maturation^23,24^, a critical event to prevent the antimicrobial function of the macrophage thus enabling mycobacterial survival and persistence. Furthermore, guinea pigs infected with the ∆*sapM* Mtb strain showed total absence of mycobacteria in lungs and spleen at 16 weeks post infection, demonstrating the critical role of SapM in Mtb pathogenesis^24^. Importantly, the lungs, liver and spleens of these animals showed only few tubercles, negligible histopathological tissue damage, and all the infected animals survived the infection^24^. Clearly, the role of SapM is important for Mtb persistence, but it also impacts on the host ability to fight the infection. Our hypothesis is that if we could block the action of SapM with a small molecule, we may be able to enhance infection clearance and help to improve TB treatments.

The *sapM* gene is unique in the mycobacterial genome and there is no human orthologue to SapM^22,25^, suggesting the potential to develop specific and selective inhibitors. Recently, we have reported that SapM can be inhibited using l-ascorbic acid (L-AC) and 2-phospho-l-ascorbic acid (2P-AC), and that inhibition reduced mycobacterial survival in human THP1 macrophages^22^. However, these are low potency inhibitors with IC_50_ values > 200 µM.

Here we have screened 1503 compounds from commercial and in-house libraries and identified three new potent (IC_50_ < 10 µM) and selective inhibitors of SapM that behave as uncompetitive inhibitors. These compounds significantly reduce up to 70% of Mtb burden in human THP-1 macrophages at a low concentration of 1 µM. Two of these inhibitors are characterised by a benzylidenemalononitrile scaffold and share a tryhydroxy-benzene moiety with the third inhibitor, a Gallic acid derivative. The most potent inhibitor, compound **1**, also reduces the burden of *Francisella tularensis* in MH-S macrophages. *F. tularensis* is the causative agent of the lethal disease tularaemia^26^ and secretes the virulence factor AcpA^27–29^, a homologue of SapM that shares the same mechanism of catalysis and inhibition^22^. Our results demonstrate the potential of exploiting antivirulence agents to impair bacterial intracellular survival for the treatment of TB, with possible applications to other microbial infections.

## Results and discussion

**Figure 1:**
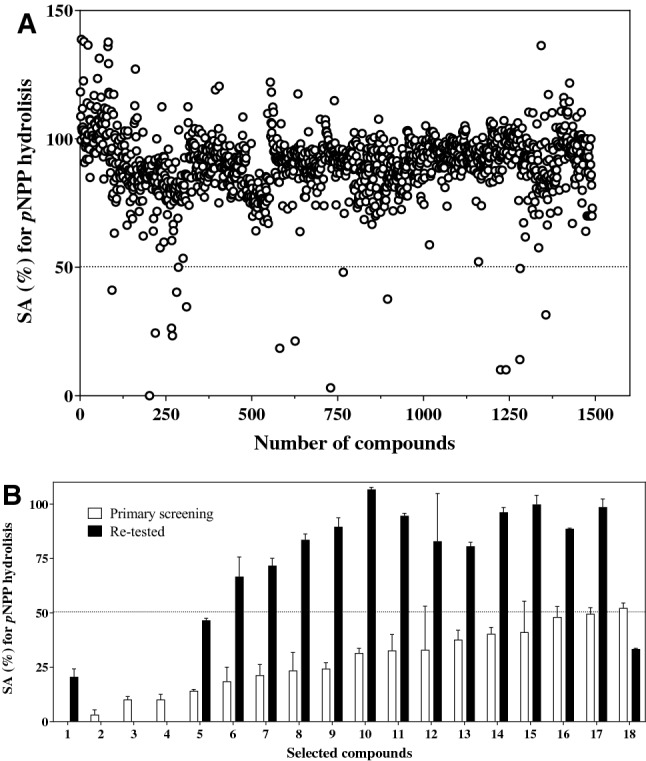
Primary screening of compounds. Enzymatic activity of SapM was assessed using the *p*NPP assay and expressed as percentage of specific activity (% SA). (**A**) Plot showing the effect of 1491 compounds at 100 µM on SapM activity (compounds ordered from left to right as LOPAC-1280, Enzo-BML-2834, LOPAC-Pfizer and in-house). Compounds that reduced SA by ≥ 50% (black dotted line) were selected. (**B**) Bar graph showing effect on SA of selected compounds from the primary screening (**A**) (bars in white) and re-tested compounds (bars in black). SA values of 0% overlap the x-axis. Percentage of SA is calculated relative to the *p*NPP DMSO control. Error bars indicate ± standard deviation of the mean (SD) of duplicates.

To identify new inhibitors of SapM, we screened four different compound libraries covering a wide range of pharmacological active drugs (LOPAC-1280 and LOPAC-Pfizer), phosphatase inhibitors (Enzo-BML-2834), and in-house compounds from other drug discovery programmes. A total of 1491 compounds were initially screened for activity against SapM. The primary screening resulted in a total of 18 compounds that reduced SapM specific activity (SA) by ≥ 50% at 100 µM (Table 1, Fig. 1A). These compounds were re-purchased and re-tested to confirm their activity. Six of the re-tested compounds showed ≥ 50% inhibition of SapM (Table 2, Fig. 1B). False positives or negatives are expected as compounds tend to degrade or precipitate after long-term storage in DMSO^30^, but the number of hits identified in our screening is similar with previous reports using the same libraries^31,32^.

**Table 1:** Summary of compounds screened from commercial and in-house libraries and structure–activity relationship (SAR) analyses.

Library	Number screened	Primary screening (≥ 50% inhibition)	Hits confirmed (≥ 50% inhibition)	IC_50_ (< 10 µM)
In-house	96	0	–	–
LOPAC-Pfizer	90	1	0	–
Enzo-BML 2834	32	3	1	0
LOPAC-1280	1273	14	5	2
SAR	12	–	4	1
Total	1503	18	10	3

**Table 2:** Hits identified in primary screen.

N	Compound name	Structure	%SA
1	Tyrphostin 51		0
2	Galloflavin		0
3	YM-26734		0
4	HI-TOPK-32		20.5 ± 3.7
5	(6R)-BH4		33.3 ± 0.3
6	RWJ-60475		46.6 ± 0.9

Percentage specific activity (SA) of compounds tested against SapM at 100 µM.

Values are average ± SD of duplicates.

The distribution of compound activity on SapM is homogeneous across the libraries (Fig. 1A), indicating an unbiased screening. Of note, some compounds increased the SA of SapM, an effect reported for allosteric activators^33^. In total, six compounds (**1**–**6**), five from the LOPAC-1280 library and one from the Enzo-BML-2834, were considered for further analyses (Table 2). These compounds are reported to target different proteins: Tyrphostin 51 and HI-TOPK-32 are kinase inhibitors^34,35^, RWJ-60475 is a tyrosine phosphatase inhibitor^36^, Galloflavin is a lactate dehydrogenase inhibitor^37^, YM-26734 is a phospholipase A2 inhibitor^38^ and (6R)-BH4, Sapropterin dihydrochloride, is a cofactor for nitric oxide synthesis^39^ used in the clinic to treat pulmonary hypertension^40^.

### Structure–activity relationship for SapM inhibitors

Among the six inhibitors, three compounds: Tyrphostin 51 (**1**), Galloflavin (**2**) and YM-26734 (**3**) completely abolished the enzymatic activity of SapM, and share the presence of a trihydroxy-benzene group (Table 2), also present in polyphenols reported as phosphatase inhibitors^41^. In order to gain structure–activity relationship (SAR) insight we searched for related compounds to **1** and **2** in the libraries tested and for commercially available derivatives. Compound **3** was not included in this search because of its complexity and poor drug-like properties according to the Lipinski’s rule of five (Ro5)^42^.

We found six additional tyrphostins related to compound **1** (**7–12** in Table S1): five from the LOPAC-1280 library (**7–11**), and one from the Enzo-BML-2834 library (**12**). All six tyrphostins showed poor activity towards SapM at 100 µM (SA > 73%). Another seven tyrphostins (**13–19**) were identified through searches in SciFinder (https://scifinder.cas.org) and tested for inhibition of SapM. Of these, **13** and **14** completely abolished enzyme activity at 100 µM (Table S1).

Additionally, nine compounds containing hydroxyl-substituted benzene rings were identified from the primary screening (**20–28** in Table S1), where **20** and **25** exhibited ~ 30% reduction in SA and the rest had poor or no activity (Table S1). A group of polyphenols found in the literature^41^ (**29–33**), including catechins and procyanidins, were tested for inhibition of SapM. Of these, **29** and **30** showed inhibition of SapM activity of about 70 and 40%, respectively (Table S1).

**Figure 2:**
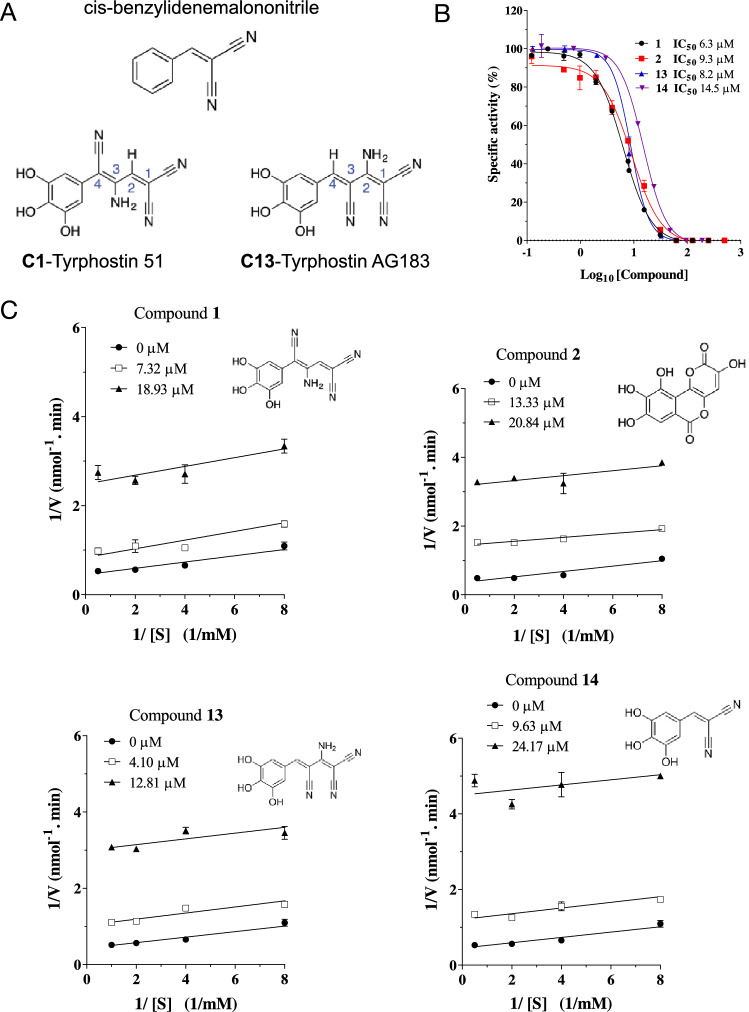
Dose–response curves and mode of inhibition for selected compounds. Compounds **1**, **2**, **13** and **14** were tested using the *p*NPP assay. (**A**) Common cis-benzylidenemalononitrile scaffold structure of the tyrphostin inhibitors and two examples compound **1** (C1) and **13** (C13). (**B**) Percentage of specific activity is calculated relative to the amount of *p*-nitrophenol released in the absence of inhibitor. Error bars represent ± SD of two independent experiments. (**C**) Mechanism of inhibition of SapM. Lineweaver–Burk plots for **1**, **2, 13** and **14**. Error bars represent ± SD of triplicates.

All tyrphostins tested (**1**, **7–19**), except **11**, contain a cis-benzylidenemalononitrile core^34^ (Fig. 2A) that mimics the moieties of tyrosine and erbstatin^43^, and one or more hydroxyl substitutions on the benzene ring, and variations in the side chain. The position of the nitrile groups or the length of the side chain, do not appear to impact on SapM inhibition. For instance, the same potency is observed for **1** and **13,** where the position of the nitrile group changes from C4 to C3, and with similar potency to **14,** with only two carbons in the side chain instead of four (Fig. 2A, Suppl. Table S1).

However, the number of hydroxyl groups (–OH) in the benzyl ring of tyrphostins, has a substantial contribution to potency because removal of one or more –OH results in loss of inhibition (Table S1). Compounds **1**, **13** and **14,** with three –OH abolished enzyme activity completely, whereas **7**, **15** and **16** with two –OH only inhibit by ~ 30–50% and **8**, **9** and **12** with one –OH inhibit by less than 21%.

For other hydroxyl-substituted benzene compounds it is clear that the presence of –OH alone is not sufficient for full potency. Compound **2** with three –OH inhibits SapM activity completely at 100 µM, but **26**, **28**, **31**–**33** with more than three –OH show none or poor activity (Suppl. Table S1). Thus, the presence of at least two –OH, appears to be necessary for inhibition particularly for the benzylidenemalononitrile scaffold, but not sufficient for full potency.

### Potency and selectivity of SapM inhibitors

IC_50_ values of key compounds showing substantial inhibition on SapM in the primary and SAR screens were determined using dose–response curves (Fig. 2B). Compound **4** was not used as it precipitates at > 100 µM, preventing the ability to reach saturation. The more potent compounds are **1**, **2**, **13** and **14**, with IC_50_ values ranging from 6 to 14 µM (Fig. 2B, Table 3). These compounds are up to 40 times more potent than the 2P-AC and L-AC inhibitors that we have previously reported (IC_50_ > 200 µM)^22^. Furthermore, all inhibitors show selectivity towards SapM over other secreted phosphatases in Mtb (Table 3): MptpB, a lipid phosphatase with a similar mode of action to SapM^18,44^ and MptpA, a tyrosine phosphatase that regulates phagosome acidification^45–47^. Importantly, compounds **1**, **2**, and **13** are also selective over the human phosphatases PTP1B (phosphotyrosine specific) and VHR (dual specificity phosphatase) (Table 3).

**Table 3:** Selectivity of SapM inhibitors towards phosphatases. Inhibition of selected compounds towards mycobacterial secreted phosphatases SapM, MptpA and MptpB, and human phosphatases PTP1B and VHR.

Compound number	IC_50_ (µM)				
	SapM	MptpA	MptpB	PTP1B	VHR
1	6.3 ± 1.0	19.8 ± 1.0	> 100	> 100	> 100
2	9.3 ± 1.1	55.2 ± 1.1	> 100	> 100	> 100
3	19.8 ± 1.1	> 100	> 100	–	–
4	ND	–	–	–	–
5	73.9 ± 1.2	–	–	–	–
6	41.9 ± 1.1	> 100	> 100	–	–
13	8.2 ± 1.0	16.7 ± 1.0	> 100	> 100	> 100
14	14.5 ± 1.0	23.7 ± 1.1	> 100	–	–
15	78.42 ± 2.7	–	–	–	–
29	63.0 ± 1.5	> 100	> 100	–	–

IC_50_ values are mean ± SD of two independent experiments.

*ND* not determined as compound precipitates at concentrations > 100 µM.

### SapM inhibitors show an uncompetitive mechanism of inhibition

Kinetics studies showed that the best four inhibitors, **1**, **2**, **13** and **14**, behave as uncompetitive inhibitors, as indicated by the characteristic pattern of parallel lines in the Lineweaver–Burk plots (Fig. 2C), where increasing amounts of inhibitor causes a reduction of both the *K*_*m*_ and *V*_*max*_ values^48^. Uncompetitive compounds bind to the enzyme–substrate complex instead of the free enzyme^48^. The potency of uncompetitive compounds is then enhanced as the substrate concentration in the reaction increases^49,50^. This mechanism is similar to the one we observed for L-AC^22^, indicating a general mechanism of inhibition for this target.

### SapM inhibitors reduce mycobacterial burden in vitro

**Figure 3:**
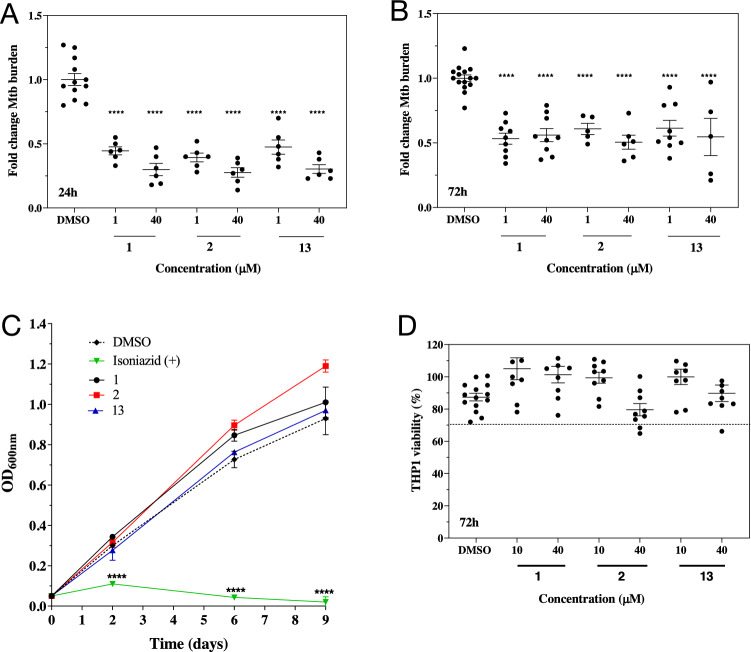
Effect of SapM inhibitors **1**, **2** and **13** on Mtb H37Rv growth and intracellular burden. Efficacy of compounds at 1 and 40 µM on Mtb *intracellular* burden in THP-1 macrophages at 24 h (**A**) and 72 h (**B**) post infection. Fold change of Mtb burden was calculated from the average CFU/ml relative to the DMSO control. Statistical significance was evaluated by one-way ANOVA (Dunnett’s test) compared to DMSO control (****p < 0.0001). Error bars in (**A,B**) indicate ± SD of two and three independent experiments, respectively. (**C**) Effect of compounds at 40 µM on the *acellular* growth of Mtb in Middlebrook 7H9 broth monitored over 9 days using optical density (OD_600_). DMSO and the first-line antibiotic isoniazid (at 0.14 µg/ml) were used as negative and positive controls, respectively. Error bars indicate ± SD of triplicates. Statistical significance was evaluated by two-way ANOVA (Dunnett’s test) compared to DMSO control (****p < 0.0001). (**D**) THP-1 macrophage viability at 72 h upon treatment with compounds at 10 and 40 µM. Percentage of viability was calculated relative to the control (RPMI media only). Dashed black line indicates 70% viability. Error bars indicate ± SD of three independent experiments.

Compounds with IC_50_ < 10 µM (Table 3) were chosen to evaluate their efficacy in THP-1 macrophages infected with the Mtb H37Rv strain. Treatment with **1**, **2** and **13** at 1 µM and 40 µM resulted in a significant (****p < 0.0001) reduction of Mtb intracellular burden in resting THP-1 macrophages at 24 h and 72 h post infection when compared to DMSO controls (Fig. 3A,B). This reduction is comparable to that observed when deleting the *sapM* gene^24^, and consistent with the critical role of SapM in intracellular mycobacterial survival^23,24^. A similar effect is observed both in vitro^12,13^ and in vivo^17,18^, when inhibiting MptpB, another secreted virulence factor involved in phagosomal maturation.

There is no substantial difference between treatment with **1** or **13**, with similar IC_50,_ suggesting that the change in the nitrile position in the benzylidenemalononitrile core does not influence efficacy in cells. Compound **2** is marginally better than **1** and **13** at 24 h but the differences are not statistically significant. The efficacy of these compounds is more pronounced at 24 h, with reductions of 50–70% on Mtb burden, compared to 30–50% at 72 h. This would agree with the proposed role of SapM in the dephosphorylation of PI(4,5)*P*_2_ and PI3*P* during phagocytosis^22^, at the initial stages of the infection. Efficacy is also superior to the one we observed for MptpB inhibitors, which showed similar reduction of Mtb burden at concentrations of 80 µM or higher, instead of 1 µM^12,18^.

None of the inhibitors tested affected acellular growth over the course of 9 days compared to the DMSO control (Fig. 3C). This is in agreement with previous reports (and our own data in Suppl. Fig. S1) that deletion of *sapM* impairs mycobacterial survival in macrophages and animal models of infection, but does not reduce growth in culture medium^24^. Furthermore, the compounds had no significant effect on the intracellular burden of ∆*sapM* (Suppl. Fig. S1)*,* supporting specificity*.*

The compounds showed no cytotoxicity (> 70% viability) up to 40 µM in THP-1 macrophages for 72 h (Fig. 3D).

Compounds **1**, **2** and **13** exhibit favourable drug-like properties (Table 4). The physico-chemical properties calculated with the SwissADME tool^51^ indicate that **1**, **2** and **13** are relatively small molecules (Mw < 300 Da), highly hydrophilic, with predicted good solubility and with lead-like properties according to the Ro5 criteria^42^. Thus this makes them suitable candidates for further development and future validation in vivo. Binding of **1**, **2** and **13** to SapM was confirmed by microscale thermophoresis (MST) (Suppl. Figs. S2, S3).

**Table 4:** Predicted physico-chemical properties of the best SapM inhibitors obtained from SwissADME^51^.

N	MW (Da)	HBA	HBD	LogP	HAC	TPSA (Å^2^)	Solubility (mg/ml)
1	268.2	6	4	− 1.38	20	158.08	5.2
2	278.2	8	4	− 0.28	20	141.34	1.3
13	268.2	6	4	− 1.38	20	158.08	5.2

*MW* molecular weight, *HBA* number of hydrogen bond acceptors, *HBD* number of hydrogen-bond donors, *LogP* partition coefficient, *HAC* heavy atoms count, *TPSA* total polar surface.

### Compound 1 reduces *Francisella tularensis* intracellular burden

One of the closest homologues of SapM is the secreted phosphatase AcpA from *Francisella tularensis*, which acts as a virulence factor for that bacterium^27,29^. We have demonstrated that SapM and AcpA share the same mechanism of catalysis^22^. We have also shown that AcpA inhibitors 2-PAC and L-AC, which reduce *F. tularensis* burden in vitro^52^, also inhibit SapM activity, and that 2-PAC significantly reduces intracellular survival of Mtb^22^. We hypothesized that SapM inhibitors may therefore have efficacy in reducing intracellular burden of *F. tularensis*. For this, the most potent compound **1** was confirmed to inhibit AcpA (Suppl. Methods and Suppl. Fig. S4) and selected to evaluate its efficacy in MH-S cells infected with *F. tularensis.*

**Figure 4:**
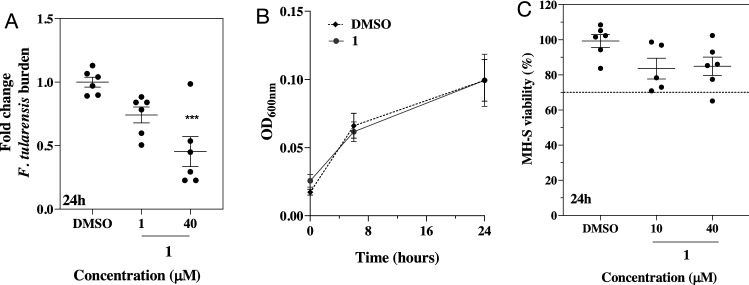
Effect of compound **1** on *F. tularensis* growth and intracellular burden. (**A**) Dose dependent efficacy of compound **1** on *F. tularensis intracellular* burden in MH-S macrophages at 24 h post infection. Bacterial burden was calculated from the average CFU/ml relative to DMSO control. Statistical significance was evaluated by one-way ANOVA (Dunnett’s test) compared to DMSO control (***p < 0.001). Error bars indicate ± SD of two independent experiments. (**B**) MH-S cells viability in percentage at 24 h post treatment with compound **1**. Percentage of viability was calculated relative to the control (RPMI media only). Dashed black line indicates 70% viability. Error bars indicate ± SD of three independent experiments. (**C**) Effect of compound **1** on the *acellular* growth of *F. tularensis* monitored over 24 h using optical density (OD_600_). Cultures were treated with 40 µM of the inhibitor. Negative control is DMSO. Error bars indicate ± SD of triplicates.

As for Mtb, compound **1** reduces *F. tularensis* intracellular burden in a dose dependent manner, with a significant reduction (55%, ***p < 0.001) at 40 μM at 24 h post infection (Fig. 4A). Compound **1** is not cytotoxic to MH-S cells (> 70% viability) (Fig. 4B), and does not affect acellular growth of *F. tularensis* (Fig. 4C). The efficacy of compound **1** in reducing Mtb and *F. tularensis* burden in infected macrophages suggests the potential of developing antivirulence agents with a broad-spectrum activity to treat microbial infections.

## Conclusions

In this study we have identified new potent inhibitors of SapM (IC_50_ < 10 µM) that have an uncompetitive mechanism of inhibition. The inhibitors are specific to SapM and selective over other Mtb secreted phosphatases and human phosphatases. The best inhibitors **1** and **13**, contain a benzylidenemalononitrile core and share a trihydroxy-benzene group with compound **2**, also found in other polyphenol phosphatase inhibitors. Importantly, the best inhibitors, show significant efficacy in reducing intracellular Mtb burden at 1 µM concentration, and recapitulate the behaviour of the ∆*sapM* strain in macrophage infections^24^ (and this study). These results are consistent with the critical role of SapM in phagosomal maturation arrest to increase Mtb survival and pathogenesis, and with the reported reduction of mycobacterial burden both in vitro^12,13^ and in vivo^17,18^, when inhibiting MptpB, another secreted virulence factor. Notably, we see also reduction in intracellular burden of *F. tularensis* when using compound **1**, suggesting the potential of developing antivirulence agents with a broad-spectrum activity to treat different microbial infections. This is the first report of potent inhibitors of SapM and provides a basis for further development to exploit the potential of this target in the treatment of TB.

## Materials and methods

### Production of recombinant proteins

SapM was expressed in *Escherichia coli* C41 (DE3) by auto-induction at 20 °C for 24 h as previously described^22,53^. The C-terminal his-tagged SapM was purified by nickel affinity chromatography and eluted with 50 mM HEPES, 500 mM NaCl, 0.05% sarkosyl, 200 mM imidazole and 3 mM ethylenediaminetetraacetic acid (EDTA), pH 7. MptpA and MptpB were expressed in *E. coli* BL21(DE3) at 18 °C with 0.5 mM isopropyl-β-d-thiolgalactopyranoside (IPTG) for MptpB and 0.1 mM IPTG for MptpA for 16 h^18^. MptpA and MptpB were both purified by nickel affinity chromatography followed by size exclusion in a Superdex75 (10/300) column (GE Healthcare) eluted with 20 mM Tris-Base, 150 mM NaCl and 3 mM EDTA, pH 7^18^. The full-length hPTP1B and hVHR proteins were expressed in *E. coli* BL21(DE3) at 18 °C with 0.5 mM IPTG for 16 h^18^. hPTP1B and hVHR were both purified using Glutathione Sepharose 4B beads (GE Healthcare) eluted with 20 mM HEPES, 500 mM NaCl, 5 mM dithiothreitol (DTT) and 10 mM glutathione (GSH), pH 7.

### Compound screening

Four libraries were selected for screening: (1) in-house compounds (96), (2) LOPAC-Pfizer library (90), (3) Enzo-BML-2834 library (32) and (4) LOPAC-1280 library (1273). LOPAC-Pfizer and LOPAC-1280 libraries were purchased from Sigma-Aldrich and the Enzo-BML-2834 library from Enzo Life Sciences. In addition, seven Tyrphostin analogues and five polyphenols were purchased from Enzo Life Sciences, Cambridge Bioscience or Sigma-Aldrich. Inhibition of SapM was tested using the *p-*nitrophenyl phosphate (*p*NPP) assay. A 150 µl of reaction mixture contained 0.5 µg of SapM protein in 50 mM Tris-Base, 150 mM NaCl, pH 7.5 and 100 µM of compounds, followed by the addition of *p*NPP (at its *K*_*m*_ concentration). The reaction mixture was incubated for 30 min at 37 °C and quenched with 50 µl of 1 M NaOH. Production of *p*-nitrophenol (*p*NP) was measured at 405 nm using a Multiskan Spectrum spectrophotometer (Thermo Scientific) and quantified using a *p*NP standard curve (15–2000 µM of *p*NP). Specific activity (SA) was calculated as nmol of *p*NP generated per µg of protein over the reaction time. Activity towards MptpA, MptpB, hPTP1B, and hVHR with the *p*NPP assay was performed in 50 mM Tris–Base, 50 mM Bis–Tris, 100 mM Sodium Acetate (pH 6 for MptpA and MptpB, and pH 5.5 for hPTP1B and hVHR) and incubated at 37 °C for 15 min^18^. The Michaelis–Menten parameters *K*_m_ and V_*max*_ were calculated using non-linear regression fit in GraphPad Prism 8.41. IC_50_ values were determined using inhibitor concentrations 0–500 µM and values calculated using a four-parameter non-linear regression fit in GraphPad Prism 8.41.

To determine the type of inhibition different inhibitor concentrations were selected to yield around 30% and 75% inhibition for each concentration of *p*NPP. This ensures sufficient signal to obtain accurate data while allowing a significant inhibition effect^48^. Velocity (*V*) was plotted as a function of *p*NPP concentration and fit in a Lineweaver–Burk plot (double-reciprocal) using GraphPad Prism 8.41. All assays were done in 96-well microplates and performed in triplicate in at least two independent experiments.

### Bacterial and cell culture

THP-1 monocyte cell lines (ATCC) were cultured in Roswell Park Memorial Institute-1640 medium (RPMI) (R8758-Sigma-Aldrich) containing l-glutamine supplemented with 10% heat inactivated fetal bovine serum (FBS, Invitrogen) at 37 °C in 5% CO_2_. Mtb strain H37Rv was grown in Middlebrook 7H9 broth (BD Diagnostics) or on Middlebrook 7H10 agar, both supplemented with 0.05% Tween 80, 0.2% glycerol and 10% OADC (Oleic Albumin Dextrose Catalase from Becton Dickinson Microbiology Systems) at 37 °C in 5% CO_2_. Mtb cultures were prepared using 1 ml of mid-log phase Mtb stock into 20 ml of fresh media and incubated static for 6 days prior to being used in infection or acellular assays.

### Cytotoxicity assays

A colorimetric assay using the tetrazolium dye 3-(4,5-dimethylthiazol-2-yl)-2,5-diphenyltetrazolium bromide (MTT) was performed as described previously^54^. THP-1 monocytes cell lines were seeded in 96-well cell culture plates, flat bottom (Corning) at a density of 5 × 10^4^ (in 200 μl media) and treated with 200 nM of phorbol 12-myristate-13-acetate (PMA) for 2 h, then media was replaced with fresh RPMI (containing 10% FBS) and cells incubated overnight. The following day media was replaced with fresh RPMI (containing 10% FBS) with 10 and 40 µM of compound **1**, **2** or **13**. This was repeated at 24 h, and at 48 h cells were washed with Dulbecco’s phosphate buffered saline (PBS) and fresh RPMI without inhibitors added. For the MH-S cells (ATCC, CRL-2019), an SV40-transformed alveolar macrophage cell line, 6 × 10^3^ cells (in 200 μl media) were seeded in 96-well cell culture plates and incubated overnight. Treatment was performed only for 24 h. Cell viability was assessed at 24 h (MH-S) or 72 h (THP-1) by adding MTT (5 mg/ml in PBS) and incubated 2 h at 37 °C in 5% CO_2_. Media was removed followed by addition of 200 µl of dimethyl sulfoxide (DMSO) and 25 µl of Sorensen’s glycine buffer, and absorbance measured at 570 nm. Each assay was performed in triplicate in at least three independent experiments. A compound was considered toxic when macrophage viability was < 70%.

### *Mycobacterium tuberculosis* infection assay

THP-1 monocytes were seeded in 24-well cell culture plates, flat bottom (Corning) at a density of 1 × 10^5^ cells per well (in 500 µl media) and treated with 200 nM PMA for 2 h, then media was replaced with fresh RPMI (with 10% FBS) and cells incubated overnight. The following day, media was replaced with fresh RPMI containing compounds at 1 and 40 µM dissolved in DMSO. Cells were infected with a multiplicity of infection (MOI) of 5:1 (bacteria:macrophage). After 4 h of infection, THP-1 cells were washed three times with PBS and fresh RPMI was added containing the inhibitors, and this was repeated at 24 h. At 24 h or 72 h, cells were lysed with 400 µl of ice-cold distilled water and together with the cell-pelleted supernatants were plated onto 7H10 agar. All experimental points were plated as tenfold dilutions in triplicate in at least two independent experiments. Colonies were counted after 14 days. Data is plotted as fold change of Mtb burden calculated from the average CFU/ml at 24 h or 72 h. A negative control of DMSO was included. Statistical significance was evaluated by one-way ANOVA followed by a multiple comparison analyses of variance by Dunnett’s test (GraphPad Prism 8.41 for Windows). Differences were considered significant at the 95% level of confidence. All experiments with Mtb were carried out in a biosafety level 3 containment facility. Note that resting macrophages were used in the Mtb infections to mimic a susceptible host (where activation may be impaired).

### *Francisella tularensis* infection assay

*Francisella tularensis* live vaccine strain (LVS), belonging to subspecies *holarctica* was used as described^55^. Briefly, to achieve the inoculum *F. tularensis* from a blood cysteine glucose agar (BCGA) plate grown for 48 h at 37 °C was resuspended in complete L-15 media (10% FCS, 5 mM l-Glutamine) (Life Technologies) to a 600 nm spectrophotometer optical density (OD) reading of 0.20 which corresponded to ~ 1 × 10^9^ CFU/ml. MOI of 100 was achieved through serial dilutions and inoculum determined by plate count on BCGA.

MH-S cells (ATCC, CRL-2019), an SV40-transformed alveolar macrophage cell line, were incubated for 2 h with the *F. tularensis* MOI 100 (bacteria:macrophage) at 37 °C to allow for cellular uptake of the bacteria. This was in the presence or absence of compound **1** (1 and 40 μM) and DMSO control. *F. tularensis* was removed and cells were washed with warm PBS, followed by 30 min incubation with 10 μg/ml gentamicin (Sigma-Aldrich) to kill any extracellular bacteria and then replaced with complete L-15 with 2 μg/ml gentamicin and compound **1** (1 or 40 μM) or DMSO control for 24 h. At 24 h cells were lysed with 4 °C water to determine bacterial burden. Statistical significance was evaluated by one-way ANOVA followed by a multiple comparison analyses of variance by Dunnett’s test (GraphPad Prism 8.41 for Windows). Differences were considered significant at the 95% level of confidence. All experiments with *F. tularensis* were carried out in a biosafety level 3 containment facility.

### Acellular growth of *Mycobacterium tuberculosis* and *Francisella tularensis*

Mtb (4.4 × 10^7^ CFUs) was cultured in 25 ml of Middlebrook 7H9 and compounds **1**, **2** or **13** at 40 μM were added on day 0. Cultures were grown static over 9 days at 37 °C in 5% CO_2_. Controls were DMSO only and isoniazid at 0.14 µg/ml. *F. tularensis* (1 × 10^8^ CFUs) was cultured in complete L-15 media (10% FCS) at 37 °C for 24 h in the presence or absence of compound **1** at 40 μM. Bacterial growth was monitored by OD at 600 nm. Experiments were performed in triplicate on at least two separate studies. Statistical significance was evaluated by two-way ANOVA followed by multiple comparison analyses of variance by Bonferroni test (GraphPad Prism 8.41 for Windows). Differences were considered significant at the 95% level of confidence.

## Supplementary Information

Supplementary Information.
