# Supplementary material for: Discovery of uncompetitive inhibitors of SapM that compromise intracellular survival of Mycobacterium tuberculosis
Source: Sci Rep. 2021 Apr 7;11:7667. doi: 10.1038/s41598-021-87117-x (PMC8027839; doi:10.1038/s41598-021-87117-x)
Supplement: Supplementary file 1 — Supplementary Information. [file 41598_2021_87117_MOESM1_ESM.docx]

**Supplementary Information**

**Discovery of uncompetitive inhibitors of SapM that compromise intracellular survival of *Mycobacterium tuberculosis*.**

Paulina Fernández-Soto^1^, Joshua Casulli^1,2,3^, Danilo Solano-Castro^1^, Pablo Rodríguez-Fernández^1^, Thomas A. Jowitt^1,3^, Mark A. Travis^1,2,3^, Jennifer S. Cavet^1^ and Lydia Tabernero^1,2,*^

^1^School of Biological Sciences, Faculty of Biology Medicine and Health, University of Manchester, Manchester Academic Health Science Centre, Manchester, M13 9PT, UK.

^2^Lydia Becker Institute for Immunology and Inflammation, University of Manchester, UK.

^3^ Wellcome Centre for Cell-Matrix Research, University of Manchester, UK.

* corresponding author: Lydia Tabernero (Lydia.Tabernero@manchester.ac.uk)

**Supplementary Methods**

***Expression and purification of the acid phosphatase (AcpA) from Francisella tularensis***

In brief, the synthetic DNA containing residues 21 to 514 from AcpA (Uniprot A0Q436) were cloned into pET-20b(+) (GenScript). DNA sequencing confirmed the inserts were in-frame and that no additional changes were introduced. The N-terminal His_6_-tagged AcpA construct was introduced into *Escherichia coli* C41 (DE3) and express by auto-induction at 20 °C for 24 h. AcpA was purified by Ni-NTA Agarose beads (Invitrogen) followed by size exclusion in a Superdex75 (10/300) column (GE Healthcare) and eluted with 20 mM Tris-Base, 150 mM NaCl and 3 mM EDTA, pH 7.

***Activity and inhibitory assays using the Malachite Green Assay***

The specific activity of AcpA towards the substrate *p*NPP was determined with the Malachite Green assay using a 100 µl reaction mixture containing reaction buffer (50 mM Tris-Base, 50 mM Bis-Tris and 100 mM sodium acetate, pH 6), 1 mM of *p*NPP and 0.1 µg of protein. The reaction mixtures were incubated at 37 °C for 30 min prior to the addition of 15 µl of malachite green reagent. The absorbance was read at 620 nm using a Multiskan Spectrum spectrophotometer (Thermo Scientific). The concentration of free phosphate produced was determined using a phosphate standard curve (25 – 3000 pmol of Sigma-Aldrich phosphate standard solution). To measure the inhibitory activity of compound **1** the reaction was incubated for 30 min at room temperature, followed by the addition of *p*NPP to a final concentration equal to its *K_m_* value and incubated for 30 min at 37 °C. However, a dose dependant curve to evaluate IC_50_ value was not performed due to compound **1** precipitation at concentrations ≥ 2 mM, which prevented the ability to reach saturation. For the inhibitory activity of 2-phospho-L-ascorbic acid (2P-AC) the *p*NPP assay was used.

***Microscale thermophoresis of SapM binding to compounds 1, 2 and 13***

Microscale thermophoresis (MST) was used to study the direct binding interaction of SapM with three compounds **1**, **2** and **13**. SapM was labelled with NTA-647nm dye which attaches to the 6-histidine tag. 100 nM NTA-647 was incubated with 200 nM SapM for 1-hour at 4 ^o^C and then centrifuged at 20,000 rcf for 20 minutes at 4 ^0^C in 20 mM HEPES, 200 mM NaCl pH 7.0. 10 mM compound was diluted to 500 μM in the same buffer. Binding checks were carried out with labelled SapM (5 nM) with and without 250 nM compound with LED power of 20% and a medium MST power in a Monolith Pico instrument and using standard capillaries. Binding analysis was performed with a doubling serial dilution from 250 nM with 16 dilutions and a constant concentration of 5 nM labelled SapM.

**Infections with SapM KO strain.**

*M. bovis* BCG Pasteur strain 1721 (streptomycin resistant, RpsL, K43R) and its *sapM* transposon insertion mutant (TnInsertion-7433) were acquired from the BCCM/ITM Mycobacteria Collection (Antwerpen, Belgium). The mutant strain was grown in the presence of 50 µg/mL of kanamycin. The insertion was verified by genomic DNA extraction and PCR. Raw 264.7 cell line macrophages were seeded in 24-well cell culture plates, flat bottom (Corning), at a density of 3x10^4^ cells per well (in 500 µl media) in Dulbecco’s Modified Eagle’s Medium–high glucose (DMEM) with 10% FBS and incubated overnight. The following day, media was replaced with fresh DMEM containing compounds at 40 µM dissolved in DMSO. Cells were infected with a multiplicity of infection (MOI) of 1:1 (bacteria:macrophage). After 4 h of infection, Raw 264.7 cells were washed three times with PBS and fresh DMEM was added containing the inhibitors, and this was repeated at 24h. At 24 h or 72h, cells were lysed with 400 µl of ice-cold distilled water and together with the cell-pelleted supernatants were plated onto 7H11 agar. All experimental points were plated as 10-fold dilutions in triplicate in at least three independent experiments. Colonies were counted after 14-21 days. A negative control with DMSO was included. Statistical significance was evaluated by two-way ANOVA with GraphPad Prism 8.41 for Windows. Differences were considered significant at the 95% level of confidence.

**Supplementary Table S1**. Percentage specific activity (SA) of compounds tested against SapM at 100 µM. Values are average ± SD of duplicates.

| **N** | **Compound**  **name** | **Structure** | **%SA** |
| --- | --- | --- | --- |
|  |  |  |  |
|  |  |  |  |
| **Tyrphostins from screen libraries** | | | |
| 7 | Tyrphostin 23 | 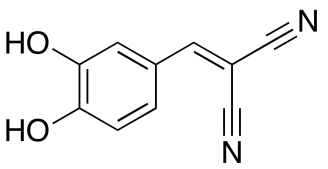 | 73.5 ± 3.1 |
| 8 | Tyrphostin AG 879 | 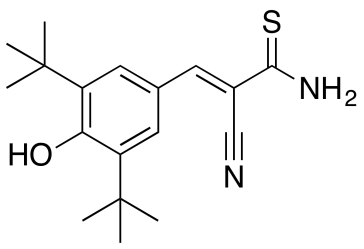 | 78.7 ± 6.6 |
| 9 | Tyrphostin AG 112 | 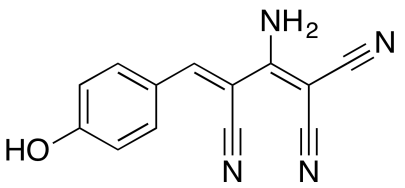 | 88.7 ± 1.7 |
| 10 | Tyrphostin 1 | 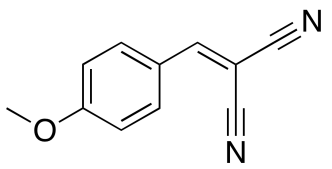 | 95.3 ± 0.9 |
| 11 | Tyrphostin AG 538 | 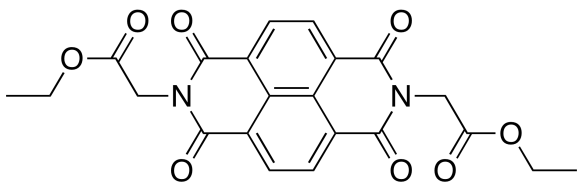 | 100.2 ± 0.3 |
| 12 | Tyrphostin 8 | 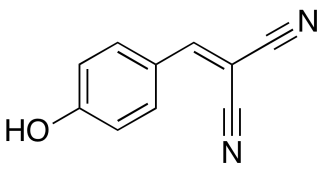 | 104.2 ± 4.2 |
| **Tyrphostins from SciFinder** | | | |
| 13 | Tyrphostin AG 183 | 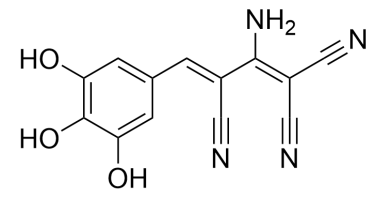 | 0 |
| 14 | Tyrphostin AG 82 | **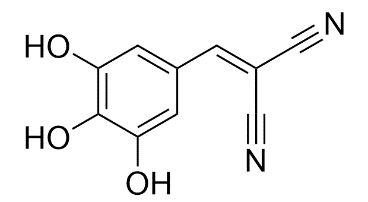** | 0 |
| 15 | Tyrphostin AG 114 | 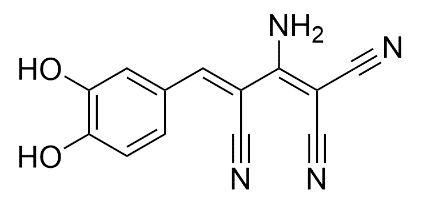 | 48.6 ± 1.1 |
| 16 | Tyrphostin AG 99 | 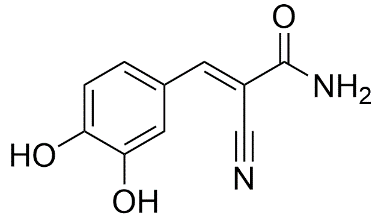 | 58.3 ± 0.6 |
| 17 | Tyrphostin AG 974 | 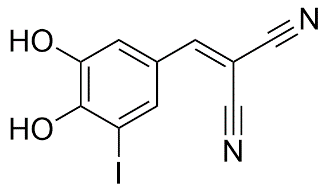 | 71.9 ± 1.4 |
| 18 | Tyrphostin AG 370 | 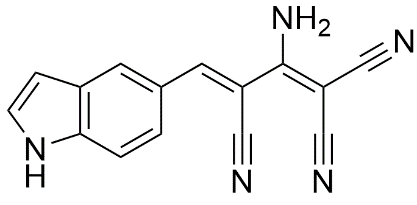 | 94.6 ± 3.1 |
| 19 | Tyrphostin AG 126 | 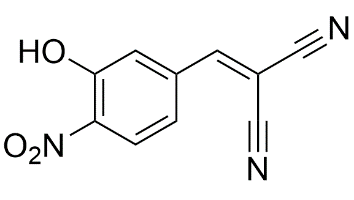 | 99.0 ± 5.5 |
| **Compounds with hydroxy-substituted benzene rings from screen libraries** | | | |
| 20 | '6-Hydroxy-DL-DOPA | 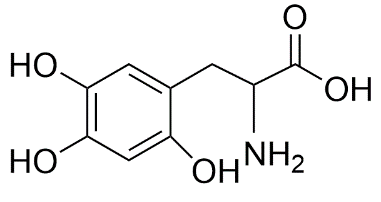 | 71.7 ± 3.4 |
| 21 | Shikonin | 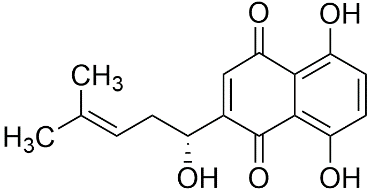 | 88.9 ± 4.3 |
| 22 | Apigenin | 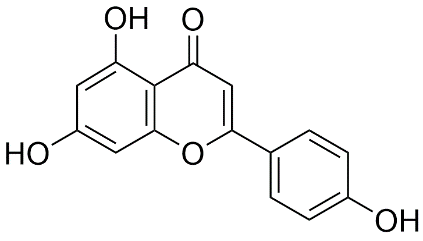 | 103.2 ± 1.1 |
| 23 | Daidzein | 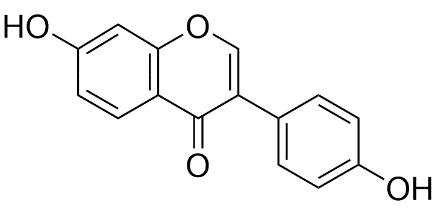 | 108.4 ± 17.2 |
| 24 | Genistein | 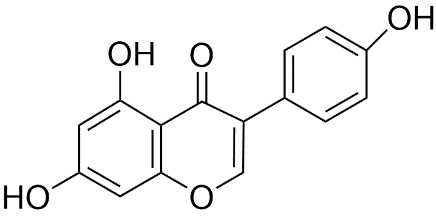 | 84.1 ± 6.4 |
| 25 | Emodin | 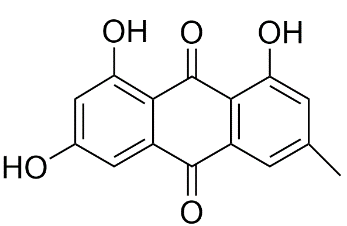 | 68.6 ± 7.2 |
| 26 | Myricetin | 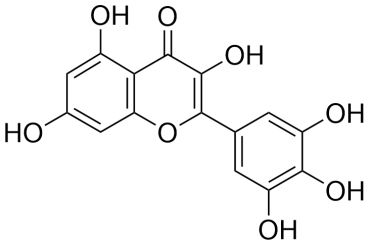 | 84 ± 6.2 |
| 27 | L-DOPS | 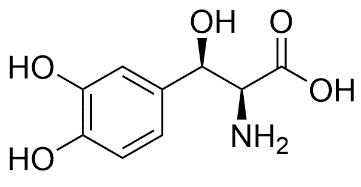 | 85.9 ± 4.2 |
| 28 | Quercetin dehydrate | 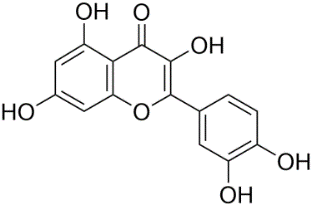 | 78.9 ± 9.2 |
| **Polyphenols from published literature** | | | |
| 29 | Gallic acid | 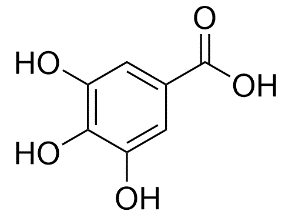 | 30.7 ± 3.6 |
| 30 | (−)-Gallocatechin | 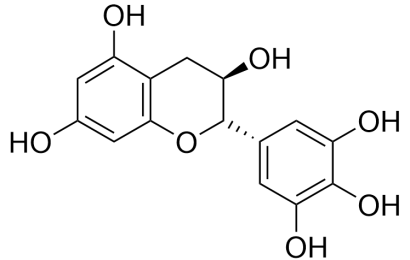 | 60.4 ± 6.4 |
| 31 | Procyanidin B2 | 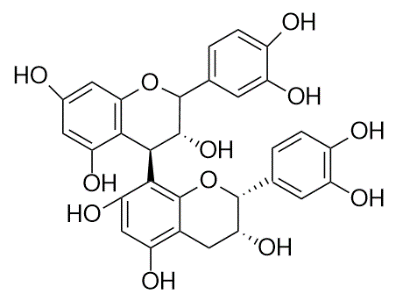 | 100 ± 14.5 |
| 32 | (−)-Epicatechin | 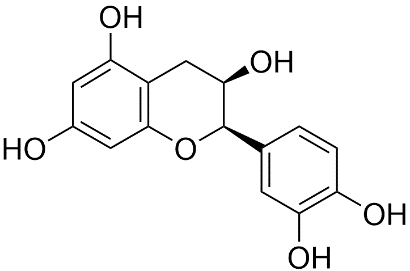 | 97.9 ± 7.2 |
| 33 | (+)-Catechin | 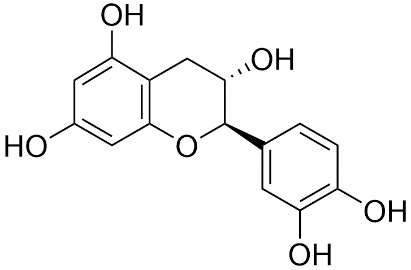 | 95.9 ± 12.3 |

**Supplementary Figure S1.**

**A**

**B**

**Figure S1.** Survival of wild-type and Δ*sapM* *M. bovis* BCG in RAW264.7 macrophages in the presence and absence of inhibitors. **A**. Intracellular burden of wild-type and Δ*sapM* BCG in the presence of DMSO (no inhibitors) at 24 and 72 hours post infection. **B.** Effect of compounds **1** or **2** (40µM, dissolved in DMSO) on the intracellular burden of *M. bovis* BCG wild-type or Δ*sapM* at 72h post-infection. The mean of three independent experiments is indicated in the plot ± SD. Statistical significance was evaluated by two-way ANOVA. Significances are shown compared to wt (_****_ *p*< 0.0001)(**A**), or to DMSO (_**_ *p*< 0.01)(**B**).

The intracellular burden of *M. bovis* BCG Δ*sapM* was significantly reduced compared to the wild-type strain at 72 hours post infection (*p*<0.0001) (Fig. S1A). Treatment with compound **1** or **2** reduced the intracellular burden of macrophages with the wild-type bacteria at 72 hours in a significant manner (*p*=0.0091 and *p*=0.0062 respectively), but did not further reduce the intracellular burden of macrophages infected with Δ*sapM* (Fig. S1B).

**Supplementary Figures S2 and S3.**


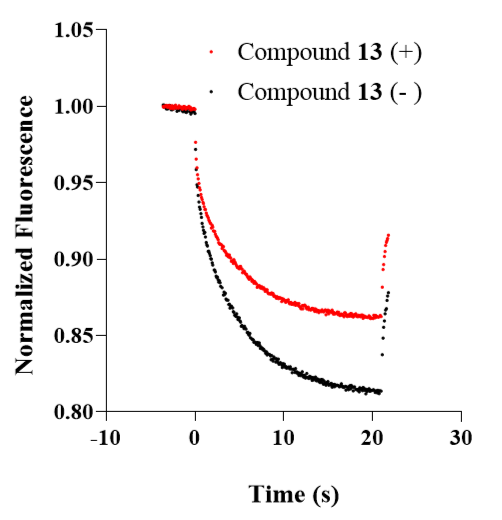

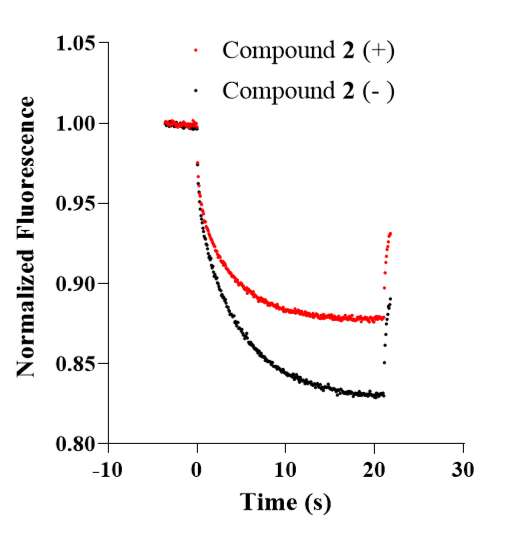

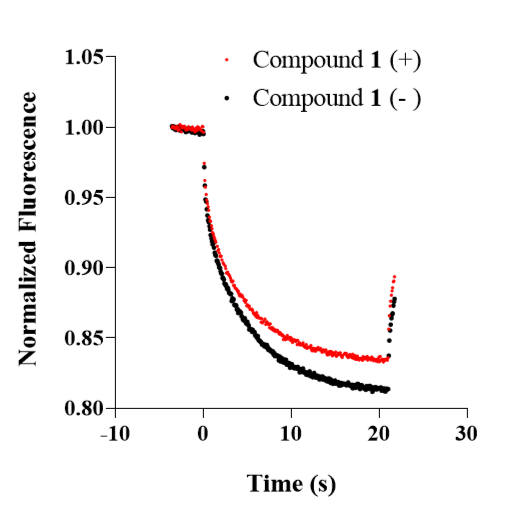


**C**

**B**

**A**

**Figure S2.** Binding change in the Microscale thermophoresis (MST) signal induced by addition of compounds 1 (**A**), 2 (**B**) and 13 (**C**). All three compounds induce a significant change in the MST signal confirming direct binding.

Binding analysis of compounds 1,2 and 13, using microscale thermophoresis (MST), showed that all three compounds produced a titratable binding response (Fig. S3). The response however was biphasic in nature with a tighter binding mode with a EC50 of approximately 0.5 μM in each case, and a weaker binding ranging from 4-60 μM (Fig. S3D), although the precise plateau for the weaker binding response is slightly ambiguous and could not be determined due to the high concentrations of compound needed to reach saturation.


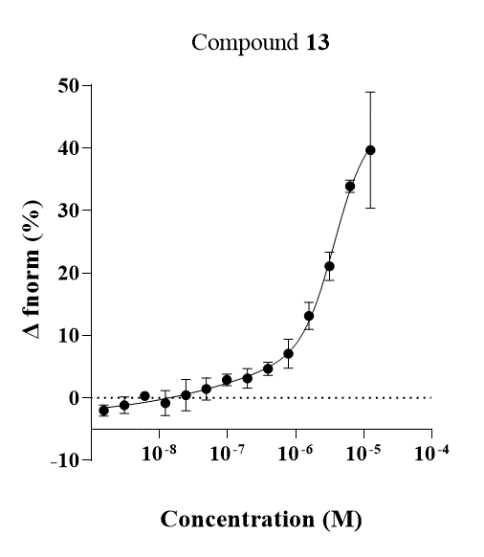
**
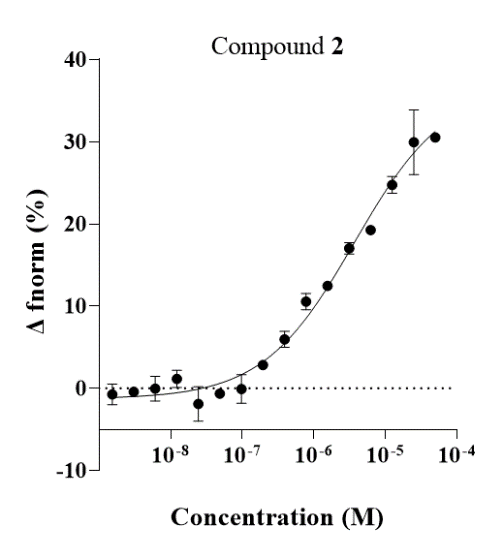
**

**A**

**B**

**C**


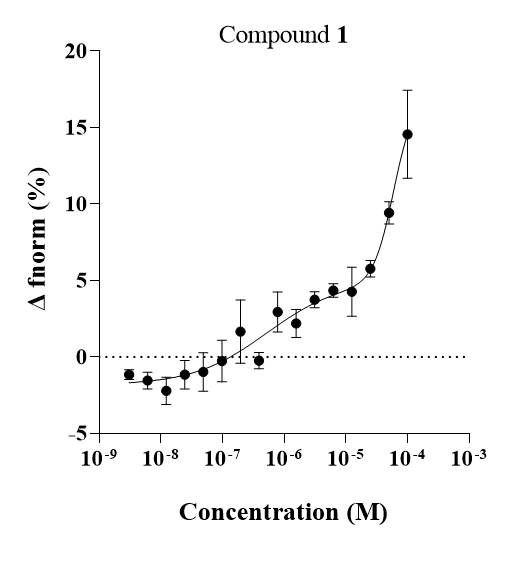


**D**

| **N** | **Single species dose response EC50 (μM)** | **Bivalent fit EC50 (1) (μM)** | **Bivalent fit EC50 (2) (μM)** |
| --- | --- | --- | --- |
| 1 | 4.6 ± 1.6 | 0.52 | 59 |
| 2 | 32 ± 16 | ----- | ----- |
| 13 | 42 ± 18 | 0.59 | 3.7 |

**Figure S3.** Titration binding curves for compounds 1 (**A**), 2 (**B**) and 13 (**C**). Microscale thermophoresis (MST) titrations fitted to a bivalent dose response model. **D.** EC50 values obtained for compounds 1, 2 and 13.

**Supplementary Figure S4.**


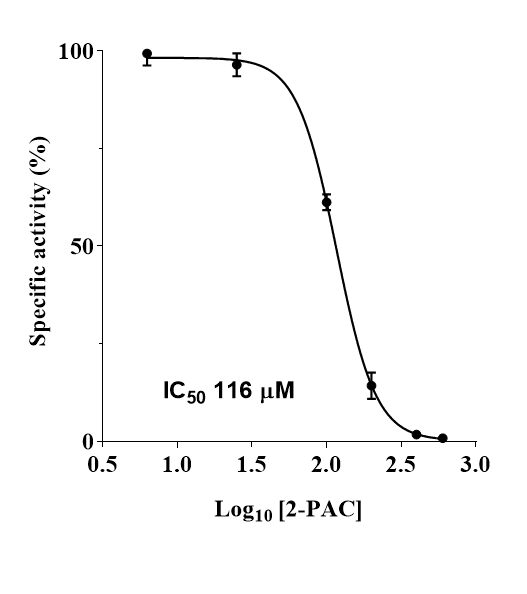

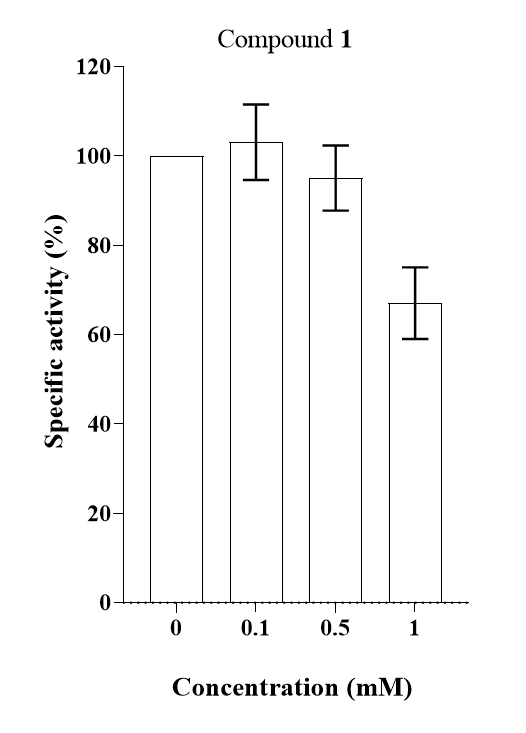


**B**

**A**

**Figure S4.** Inhibition and dose-response curve of the enzymatic activity of AcpA expressed as percentage of specific activity (%SA). **A.** Bar graph showing the effect on SA of compound 1 using the Malachite Green assay. **B.** Inhibition curve for and 2-phospho-L-ascorbic acid (2P-AC) using the pNPP assay. Percentage of specific activity is calculated relative to the amount of *p*-nitrophenol released in the absence of inhibitor. Error bars represent ± SD of triplicates.

Compound **1** inhibited the enzymatic activity of AcpA around 30% at a concentration of 1 mM (Fig. S4A). Compound 2-phospho-L-ascorbic acid (2P-AC), an inhibitor of AcpA previously reported^52^, was used as a positive control. 2-PAC inhibited of AcpA with an IC_50_ of 116 ± 1.02 µM (Fig S4B).
